# Bioactive gliadin electrospinning loaded with *Zataria multiflora Boiss* essential oil: Improves antimicrobial activity and release modeling behavior

**DOI:** 10.1002/fsn3.3062

**Published:** 2022-11-09

**Authors:** Zohreh Bahrami, Ahmad Pedram‐Nia, Mohammadreza Saeidi‐Asl, Mohammad Armin, Mojtaba Heydari‐Majd

**Affiliations:** ^1^ Department of Food Science and Technology, Sabzevar Branch Islamic Azad University Sabzevar Iran; ^2^ Department of Agronomy and Plant Breeding, Sabzevar Branch Islamic Azad University Sabzevar Iran; ^3^ Department of Nutrition, Research Center for Clinical Immunology Zahedan University of Medical Sciences Zahedan Iran

**Keywords:** electrospinning, gliadin, kinetics of release of nanofiber mats, physicochemical properties, *Zataria multiflora* essential oil

## Abstract

This study aimed to produce electrospun gliadin nanofibers containing *Zataria multiflora* Boiss essential oil (ZMEO) (5, 10, and 15% w/w), thereby developing active, sustained‐release antimicrobial mats. By increasing the level of the ZMEO, the zeta potential and electrical conductivity increased, but the viscosity and consistency index decreased. All feed solutions demonstrated shear‐thinning behavior, and the power law model was the best model. Field emission scanning electron microscopy (FESEM) images proved that the gliadin nanofibers showed a uniform, beaded‐free structure at different levels of ZMEO, with an average diameter of between 403.87 ± 15.29 and 522.19 ± 11.23 nm. Increments in the level of ZMEO decreased the mats' tensile strength and Young's modulus but increased their elongation at break. Fourier transform infrared (FTIR) and differential scanning calorimetry (DSC) analysis confirmed that the ZMEO was well loaded within these structures, augmenting its thermal stability. The studied Gram‐negative bacteria (*Escherichia coli and Pseudomonas aeruginosa*) were more resistant to the ZMEO than the Gram‐positive bacteria (*Bacillus cereus and Staphylococcus aureus*). The Peleg model was the most suitable model for describing the ZMEO release behavior, the mechanism of which was primarily Fickian diffusion.

## Graphical abstract

Electrospinning‐assisted fabrication of gliadin was performed. The effects of processing parameters on morphology and properties were studied. The *Zataria multiflora Boiss* essential oil (ZMEO) was electro‐encapsulated in the nanofibers. Kinetic modeling on the ZMEO release from gliadin nanofibers was applied. Different release behaviors of the ZMEO were studied.

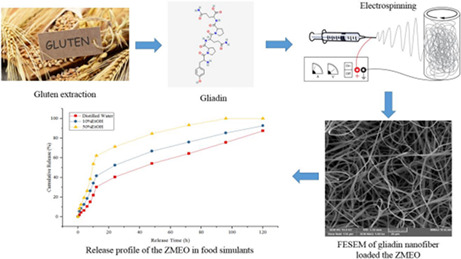

## 1 INTRODUCTION

Food contamination, caused by microorganisms, would shorten the shelf‐life of food and contribute toward waste, as well as, increase the risk of foodborne illness (Salarbashi et al., 2020). Hence, it is therefore imperative in the food packaging industry for applying effective methods to inhibit or reduce foodborne pathogenic microorganisms on the surface of food products and promoting quality and safety. Recently, the development of science and nanotechnology has brought new advanced food packaging with antimicrobial properties to reduction in wastage and increase the shelf‐life of food (Yong & Liu, 2021). Active packaging is an emerging and existing area of food technology that intentionally releases or absorbs nutritional and medicinal compounds from the food or the headspace of food packaging, which provide better food preservation by stalling the undesirable reactions of lipid oxidation, microbial growth, and moisture loss and gain better than traditional food packaging (Firouz et al., 2021). Nano antimicrobial packaging is one of the most promising active packaging concepts which can extend the shelf‐life of foods through a mild preservation technique (Yong & Liu, 2021). The inclusion of active ingredients into nonfunctional packaging systems (e.g., fibers, particles, wraps, or films) has been shown to enhance the shelf‐life of foods. One of the most important systems for carrying nutrients is nanofibers (Heydari‐Majd, Ghanbarzadeh, Shahidi‐Noghabi, Najafi, & Hosseini, 2019).

The nanofibers (with a diameter of around 100 nm or less) are porous and consist of a sheet‐like network of amorphous fibers with specific features such as large specific surface area, unique flexibility, and high density of nanometer‐sized pores of the mat (Heydari‐Majd, Rezaeinia, Shadan, Ghorani, & Tucker, 2019). These features make nanofibers more reactive toward multiple fields, including drug delivery, controlled release systems, and personal care. One of the latest methods for nanofiber production, which is used to protect sensitive compounds such as essential oils (EOs), is the electrospinning process (Rezaeinia et al., 2019; Rezaeinia, Ghorani, et al., 2020).

Electrospinning is one of the novel encapsulation techniques for the production of ultrafine fiber mats ranging from several tens of nanometers using high voltages from a precursor polymer solution. The technique involves the pumping of a polymer solution with bioactive compounds from the needle jet to the collector, applying high‐voltage electrical force between the tip of the needle and the collector which results in the production of micron, submicron, and nanofiber mats on the collector surface (Ghorani et al., 2020).

Several food‐grade polymers are used to encapsulate active pharmaceutical and food compounds in electrospinning techniques. Among these polymers, gliadin has the ability to encapsulate and control the release of diverse bioactive molecules (Hajjari et al., 2021). Monomeric gliadins are important storage proteins in wheat gluten. Gliadin (a type of storage prolamin in wheat kernels) is the water‐insoluble and amphiphilic component of gluten and consists of glycoproteins rich in proline and glutamine. Gliadins play an important role in the development of food‐grade delivery systems because they are highly hydrophobic and have bioadhesive properties through hydrogen bonding and electrostatic interactions to the intestinal membrane, a fact of outmost importance for fabricating electrospun fiber mats for various food applications (Sharif et al., 2019).

An important strategy to improve the functionality of polymers and nanofibers in packaging is to activate them with various types of naturally active compounds such as essential oils, colorants, flavors, and antimicrobials, to extending the shelf‐life of the food products (Heydari‐Majd, Rezaeinia, Shadan, Ghorani, & Tucker, 2019). Among various types of natural additives, essential oils (EOs) as used in food preservation are known as the secondary metabolites of the plants which contain high levels of polyphenols and possess antimicrobial and antioxidant properties (Bahrami et al., 2022; Heydari‐Majd et al., 2022; Monjazeb Marvdashti et al., 2022). Among EOs, *Zataria multiflora Boiss*. (Avishan‐e‐Shirazi in Persian name) is a member of the *Labiatae family* and cultivated extensively wild in the central and warm parts of Iran, Pakistan, and Afghanistan (Abdolshahi et al., 2018). The oil and extracts of *Z. multiflora* successfully have shown antibacterial (against a number of Gram‐positive and Gram‐negative bacteria) and antifungal activities. In addition, *Z. multiflora* EOs contain a high level of polyphenols that exhibit antioxidant and antimicrobial activities (Heydari‐Majd, Ghanbarzadeh, Shahidi‐Noghabi, Najafi, & Hosseini, 2019). The direct addition of EOs to food products is often accompanied by limitations such as the decomposition of these compounds by chemical processes and the alteration of the organoleptic properties of the product (Heydari‐Majd, Rezaeinia, Shadan, Ghorani, & Tucker, 2019). The main way to overcome this problem is the addition of EOs to nanofiber mats.

Although few studies on gliadin nanofibers have been reported in the literature, no reported data are available on the use of ZMEO as an antimicrobial agent in the processing of gliadin nanostructured mats. Hence, the purpose of this study was drawn to first incorporate three concentrations of ZMEO into electrospun gliadin nanofibers using the electrospinning process for enhanced antibacterial activity and then evaluate the effect of these levels of EOs' content on the morphostructural property of nanofibers, thermal properties, rheological properties, the antimicrobial activity, and interactions between gliadin and EOs. In addition, the kinetic release of ZMEO from gliadin nanofibers was investigated.

## 2 MATERIALS AND METHODS

### 2.1 Chemicals and samples

Gluten of wheat and ZMEO were kindly provided by Yas Co. (Mashhad, Iran) and Exir Gole Sorkh Co. (Mashhad, Iran), respectively. Ethanol (absolute, HPLC grade, 99.8%) was purchased from Düzey LAB Ltd., Turkey, and Mueller–Hinton Agar (MHA) was purchased from Merck, Germany. For antimicrobial tests, bacterial stocks, such as *Staphylococcus aureus* (*PTCC 25923*), *Bacillus cereus* (*PTCC 1247*), *Escherichia coli* (*PTCC 1338*), and *Pseudomonas aeruginosa* (*PTCC 1707*), were provided from the Institute of Standards and Industrial Research of Iran. All the applied reagents were of analytical grade and used as received.

### 2.2 GC–MS analysis of ZMEO

Constituents of the ZMEO were determined using gas chromatography–flame ionization detector (GC–FID) (Perkin Elmer Autosystem XL) analyses of the oil equipped with a DB‐5 fused silica column (60 m × 0.25 mm i.d.) (Heydari‐Majd, Ghanbarzadeh, Shahidi‐Noghabi, Najafi, & Hosseini, 2019). The chromatographic conditions were run under the following condition: carrier gas, helium, with a flow rate of 1.5 ml/min with a split ratio equal to 20:1; initial column temperature of 100°C for 5 min, followed by the first temperature rise up to 130°C with the gradient of 10°C/min, followed by a temperature enhancement of 7°C/min up to 270°C, and finally holding isothermal at the mentioned temperature for 3 min. The unknown compounds were identified by a comparison of their mass spectra with those of authentic samples and with available library data of the Wiley 275 MS standard database.

### 2.3 Nanofiber preparation

#### 2.3.1 Gliadin extraction and solution preparation

Gliadin extraction was performed according to the method reported by Sharif et al. (2019) with some modifications. Briefly, 5 g of gluten powder was gently stirred with 60 ml of 70% v/v ethanol (gluten powder/solvent ratio of 1/12 w/v) with a magnetic stirrer at room temperature for 4 h. Then suspensions were centrifuged at 10,000 rpm (revolutions per minute) for 15 min. Finally, gliadin was obtained from the supernatant of each step after the evaporation of ethanol.

#### 2.3.2 Electrospinning process

The gliadin extracted from gluten was dissolved at a concentration of 30% (w/v) in 70% ethanol and stirred for 15 min at 3000 rpm. Then, ZMEO with three different levels (5, 10, and 15% w/w) was added to the gliadin solution and after complete dissolution, each feed solution was allowed to rest for 20 min to completely remove air bubbles. Then, each of the prepared solutions was poured into a plastic syringe and spinning was performed using a syringe pump with a flow rate of 1.5 ml/h, equipped with a high‐voltage electric field (voltage 25 kV). The distance between the tip of the needle (G 18, Sigma‐Aldrich) and the collector (aluminum collector plate, 150 × 150 mm^2^) was maintained at 150 cm. Spinning was performed for 30 min at room temperature (25 ± 3°C) and relative humidity of 24 ± 3%.

#### 2.3.3 Solution properties

##### Electrical conductivity

To determine the electrical conductivity of each of the feed solutions of the electrospinning process, an electrical conductivity meter (model 3540, Jenway) was used with three different replications at 25°C (Wang et al., 2021).

##### Zeta potential

The surface charge of the solutions was determined by the dynamic light diffraction method using a zetasizer (Malvern Zetasizer Nano ZS, Worcestershire, UK) under ambient temperature and pH = 6.18 with 3 replications (Liu et al., 2020).

##### Apparent viscosity

Brookfield viscometer (Brookfield DVIII Ultra, Brookfield Engineering Laboratories, Stoughton, MA, USA) was used to determine the apparent viscosity of feed solutions of the electrospinning process. For this purpose, the spindle SC4‐27 was selected and the flow characteristics were determined in the shear rate range of 1–100 1/s under ambient temperature. SlideWrite Plus Graphics software (version 7.01, USA) was also used to fit the flow properties' data with experimental models of the Herschel–Bulkley (Equation 1) and Power law (Equation 2) (Rezaeinia et al., 2019).

(1) σ=σ0+kẏn

(2) σ=kẏn

where σ_0_, σ, k, ẏ, and n are the yield stress (Pa), shear stress (Pa), consistency index (Pa.sn), shear rate (1/s), and flow index (dimensionless), respectively.

#### 2.3.4 Characterization of ZMEO‐loaded gliadin nanofiber mats

##### Mechanical property

Mechanical properties, such as tensile strength (TS), elongation at break (% E), and Young's modulus (EM), were studied to evaluate the effect of the ZMEO on these indices. For this purpose, the loaded electrospun gliadin mats with dimensions of 40 × 10 mm^2^ were cut and by a Tensile Tester (TA‐XT Plus, Stable Micro Systems Ltd.) with a crosshead speed of the device grips adjusted to 1 mm/min were analyzed (Qin et al., 2017).

##### FT‐IR analysis

The infrared spectra of the gliadin samples were obtained using a Fourier transform infrared (FTIR) spectrometer (Bruker Alpha FTIR, USA) in the range of 400–4000 cm^−1^ wavenumber at a resolution of 4 cm^−1^ to investigate the molecular organization of the samples (Abdolshahi et al., 2020).

##### Morphological analysis of the gliadin structures

The nanofibers' microstructure was observed using a field emission scanning electron microscope (FESEM) (TESCAN, Czech Republic) by applying an accelerating voltage of 20 kV (Heydari‐Majd, Rezaeinia, Shadan, Ghorani, & Tucker, 2019). Before the analysis, each nanofiber was fixed onto aluminum pins using a double‐sided adhesive tape. Then the samples were gold coated using a BAL‐TEC SCD 005 sputter coater (BAL‐TEC AG, Balzers, Liechtenstein) under 15 mA for 60 s. Average fiber diameters and fiber size distributions were obtained from a minimum of 100 measurements from each image using image analysis software (Digimizer, MedCalc Software, Belgium).

##### Differential scanning calorimetry (DSC)

Thermal properties of gliadin nanofibers were measured using DSC (PerkinElmer, Akron, OH, USA) at temperatures from 35°C to 250°C at a heating rate of 10°C/min under a N_2_ atmosphere with a 50 ml/min flow rate (Sharif et al., 2019).

##### Antibacterial activity of nanofibers

Antimicrobial activity of gliadin nanofibers containing various concentrations of ZMEO on *E. coli*, *B. cereus*, *S. aureus*, and *P. aeruginosa* was investigated using the agar diffusion method and inhibition zone measurement (Aghaei et al., 2020). For this purpose, the gliadin nanofiber samples were first cut into sterile disks with a diameter of 6 mm using a sterile punch. In the following, the sterile disks were placed in microbial plates containing MHA culture medium, which had been precultured with 100 μl of overnight liquid culture approximately equivalent to 10^8^ CFU/mL of these bacteria. The cultured plates were incubated overnight at 30°C and finally, the antimicrobial property was calculated based on the inhibition zone.

##### In vitro release modeling of the ZMEO

Various food simulants were used to evaluate the release of the ZMEO in these media. Food simulants for water‐based media (distilled water), oily media (50% ethanol), and alkaline or alcoholic media (10% ethanol) were selected for this purpose. By immersing a mat with dimensions of 3 × 3 cm^2^ in each of the mentioned media, the release kinetics of the ZMEO at room temperature during 120 h (0, 1, 2, 4, 6, 8, 10, 12, 24, 48, 72, 96, and 120 h) were calculated. At each given time, 1 ml of each medium was removed and mixed with 1 ml of ethanol. After centrifuging the resulting mixture at 5000 rpm for 10 min, its absorbance at 230 nm was measured by a spectrophotometer (UNICO‐2100). It should be noted that the volume removed from the medium was replaced with fresh media solution (Alinaqi et al., 2021).

The dominant mechanism governing the release of the ZMEO from the structure of electrospun gliadin fibers in food simulants media was determined using different equations 3–7 as shown in Table 1 (Higuchi, 1963; Kopcha et al., 1990; Peleg, 1988; Peppas & Sahlin, 1989; Ritger & Peppas, 1987).

**TABLE 1:** Mathematical models for the analysis of *Zataria multiflora Boiss* essential oil (ZMEO) release kinetic from gliadin electrospun nanofibers

Model	Formula	Parameters	Reference
Higuchi	M_t_/M_∞_ = kt^1/2^	k: Release constant t: time	Higuchi (1963)
Rigter–Peppas	M_t_/M_∞_ = kt^n^	k: Release constant t: time n: Release exponent *n* ≤ 0.45: Fickian diffusion 0.45 < *n* < 0.89: Non‐Fickian diffusion *n* ≥ 0.89 up to 1: Erosion	Ritger & Peppas (1987)
Peppas–Sahlin	M_t_/M_∞_ = k_1_t^m^ + k_2_t^2m^	k_1_: Fickian constant k_2_: Erosion constant t: time m: Fickian diffusion exponent k_1_/k_2_ > 1: Mainly Fickian diffusion k_1_/k_2_ < 1: Mainly erosion k_1_/k_2_ = 1: Fickian diffusion & erosion	Peppas & Sahlin (1989)
Kopcha	Mt = A × t^0.5^ + B × t	A/B > 1: Mainly Fickian diffusion A/B < 1: Mainly erosion A/B = 1: Fickian diffusion and erosion	Kopcha et al. (1990)
Peleg	t/M_t_ = k_1_ + k_2_t	1/k_1_: Release rate 1/k_2_: ZMEO released in M∞ t: time	Peleg, et al. (1988)

### 2.4 Statistical anylysis

This study was conducted for statistical analysis using a completely randomized design with three observations. One‐way analysis of variance (ANOVA) was performed using the SPSS statistics software (version 11.5, IBM Corp.) followed by Duncan's multiple comparisons test at 5% probability level. MATLAB R2010b software (MathWorks) was also used to perform the sensitivity analysis and to solve the parameters of release with the help of nonlinear regression (nlinfit function), and finally, to generate the figures.

## 3 RESULTS AND DISCUSSION

### 3.1 Chemical composition of ZMEO

Identifying the chemical composition of the EOs is very important to justify their antimicrobial properties. The chemical composition of ZMEO is shown in Table 2. About 33 compounds were identified in the current, but 15 indicator compounds were reported. According to Table 2, O*‐Cymene* (15.70%), *Carvacrol* (8.98%), and *Thymol* (7.32%) accounted for the largest percentage of main ZMEO components, respectively. Similar research has shown that Thymol and Carvacrol were the main constituents of ZMEO (Keykhosravy et al., 2020; Nakhaee Moghadam et al., 2021). Observation of some previous reports from other researchers in relation to the main ZMEO components showed small quantitative and qualitative differences with the current study (Heydari‐Majd, Ghanbarzadeh, Shahidi‐Noghabi, Najafi, & Hosseini, 2019; Nakhaee Moghadam et al., 2021). These differences are related to differences in plant species, ecotypes, and geographical conditions (Heydari‐Majd, Ghanbarzadeh, Shahidi‐Noghabi, Najafi, & Hosseini, 2019).

**TABLE 2:** Chemical composition of *Zataria multiflora Boiss* essential oil (ZMEO)

No.	Compounds (ZMEO)	RT	%
1	α‐Pinene	9.69	5.57
2	‐o‐Cymen	14.49	15.70
3	γ‐Terpinene	15.97	4.84
4	Carvacrol methyl esther	24.72	4.03
5	m‐Thymol	27.29	5.11
6	Cresol	27.44	4.02
7	Thyme camphor	29.72	6.90
8	Thymo	27.86	7.32
9	Hydroxy‐p‐cymene l	29.99	4.96
10	Isopropyl‐5‐methylphenol	28.11	6.38
11	Isopropyl‐1‐methylbenzene	28.40	6.07
12	p‐Cymen‐2‐ol	28.53	7.22
13	Isothymol	28.57	4.21
14	Carvacrol	28.85	8.98
15	Carvone	28.98	4.19
‐	Others	‐	2.3
‐	Total	‐	97.8

Abbreviation: RT, Retention time (min).

### 3.2 Properties of solutions

Properties of solutions such as zeta potential, electrical conductivity, and apparent viscosity are among the properties that directly affect the various properties of feed solutions for electrospinning operations. In this study, the effect of using different levels of ZMEO (5, 10, and 15% w/w) on the properties of feed solutions used to electrospinning gliadin was evaluated.

Based on the results, it was found that the zeta potential of pure gliadin solution is 15.21 ± 0.12 mV, which by increasing the use of ZMEO in the preparation of the solutions, the zeta potential magnitude increased significantly (*p* < .05). Therefore, as shown in Table 3, the value of the zeta potential increased from 16.85 ± 0.14 to 21.59 ± 0.22 mV.

**TABLE 3:** Rheological parameters, electrical conductivity, and zeta potential of pure gliadin solution and gliadin solutions containing the *Zataria multiflora Boiss* essential oil (ZMEO) in the power law model

Sample	K (Pa.s^n^)	*n*	*R* ^2^	*R* ^2^‐Adj	Electrical conductivity (μS cm^−1^)	Zeta potential (mV)
Gliadin	1.64 ± 0.005^a^	0.73 ± 0.001^a^	0.99	0.99	141.68 ± 0.15^d^	15.21 ± 0.12^d^
Gliadin +5% ZMEO	1.45 ± 0.004^b^	0.64 ± 0.002^b^	0.98	0.98	152.38 ± 0.18^c^	16.85 ± 0.14^c^
Gliadin +10% ZMEO	1.30 ± 0.003^c^	0.52 ± 0.003^c^	0.99	0.99	158.15 ± 0.09^b^	18.73 ± 0.19^b^
Gliadin +15% ZMEO	1.12 ± 0.004^d^	0.41 ± 0.002^d^	0.99	0.99	167.53 ± 0.12^a^	21.59 ± 0.22^a^

*Note*: Different letters in the same column indicate significant differences (*p* < .05).

Examination of the electrical conductivity of the samples also showed that the pure solution of gliadin had an electrical conductivity of 141.68 ± 0.15 μS cm^−1^, which by using ZMEO and increasing its amount, the electrical conductivity of the solutions increased significantly from 152.38 ± 0.18 to 167.53 ± 0.12 μS cm^−1^ (*p* < .05).

**FIGURE 1:**
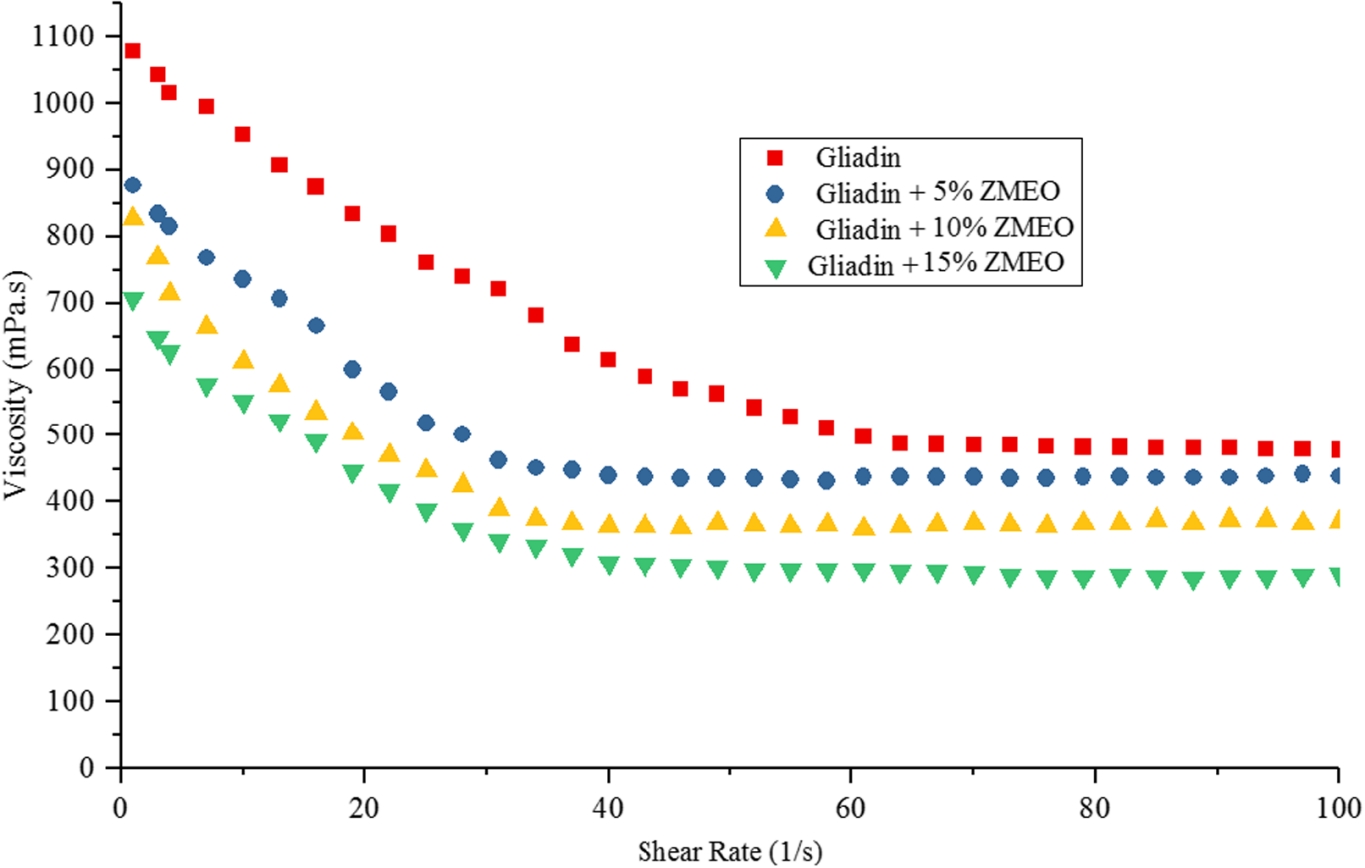
Apparent viscosity of pure giadin solution and gliadin solutions containing the *Zataria multiflora Boiss* essential oil (ZMEO)

The flow characteristics of the solutions produced as feed for the electrospinning process were also studied and fitted with Herschel–Bulkley and the power law models. The results of this analysis showed that all samples have a shear‐thinning behavior so that with increasing shear rate, the apparent viscosity of the solutions decreased. Fitting of rheological parameters with experimental models showed that the power law model had the highest coefficient of expansion (*R* ^2^ = 0.998) and the rheological data were well fitted with this model (Table 3). Accordingly, as shown in Figure 1, with increasing the level of the ZMEO application in the feed solution formulations, their viscosity decreases, so that the lowest apparent viscosity in relation to gliadin solution contains 15% w/w of the essential oil. Also, the study of rheological parameters of the power law shows that by increasing the level of the ZMEO, the consistency index (k) of the samples decreases significantly (*p* < .05) (Table 4). n is the dynamic power law exponent within the range of 0–1, in which *n* = 1 represents the elastic state and *n* = 0 indicates the viscous state. The flow behavior index n of all the solutions was less than 1.0, suggesting a non‐Newtonian fluid with shear‐thinning property, which is an important requirement for injectable materials (Table 3). It has been reported that a weak shear‐thinning behavior is necessary for biopolymer electrospinning (Kutzli et al., 2019). The higher shear‐thinning degree of biopolymer solutions causes lower viscosity with increasing shear rate of the jet stream. The high viscosity can lead to (1) attenuation of electrical charge, (2) probable syringe blockage, and (3) limits usable biopolymer concentration for required polymer chain entanglement. So that, higher shear‐thinning properties of biopolymer solution can be useful for the electrospining of them (Fang et al., 2011). These behaviors are probably due to the plasticizing ability of the EOs. The ZMEO, due to its plasticizing ability, is placed between biopolymer chains, and these chains easily slide together as a result of shear force. Therefore, with increasing the level of essential oil application, this ability increases, which is accompanied by a decrease in the viscosity of feed solutions. Also, the shear‐thinning behavior can be related to the alignment of gliadin biopolymer chains with the shear force applied to the solutions (Karim et al., 2021). The results of changes in the viscosity of feed solutions were consistent with the results of other researchers. Based on the results presented by Vafania et al. (2019) on changes in the flow behavior of chitosan–gelatin feed solutions containing thyme for the production of nanofibers by nozzle‐less electrospinning, it was found that increasing the level of essential oil application reduces the viscosity of solutions. Decreased viscosity and the shear‐thinning behavior by shear force these solutions were attributed to the plasticizing ability of the essential oil.

**TABLE 4:** Mechanical properties, elongation at break (%E), Young's modulus (EM), and tensile strength (TS), gliadin‐based mats

Sample	E (%)	EM (MPa)	TS (MPa)
Gliadin	8.21 ± 1.23^d^	32.41 ± 2.10^a^	7.23 ± 1.18^a^
Gliadin +5% ZMEO	12.10 ± 1.12^c^	24.12 ± 1.32^b^	5.36 ± 1.15^b^
Gliadin +10% ZMEO	16.09 ± 1.13^b^	19.38 ± 2.11^c^	3.14 ± 1.11^c^
Gliadin +15% ZMEO	20.36 ± 1.21^a^	15.52 ± 1.14^d^	1.03 ± 1.06^d^

*Note*: Different letters in the same column indicate significant differences (*p* < .05).

### 3.3 Morphological characteristics

**FIGURE 2:**
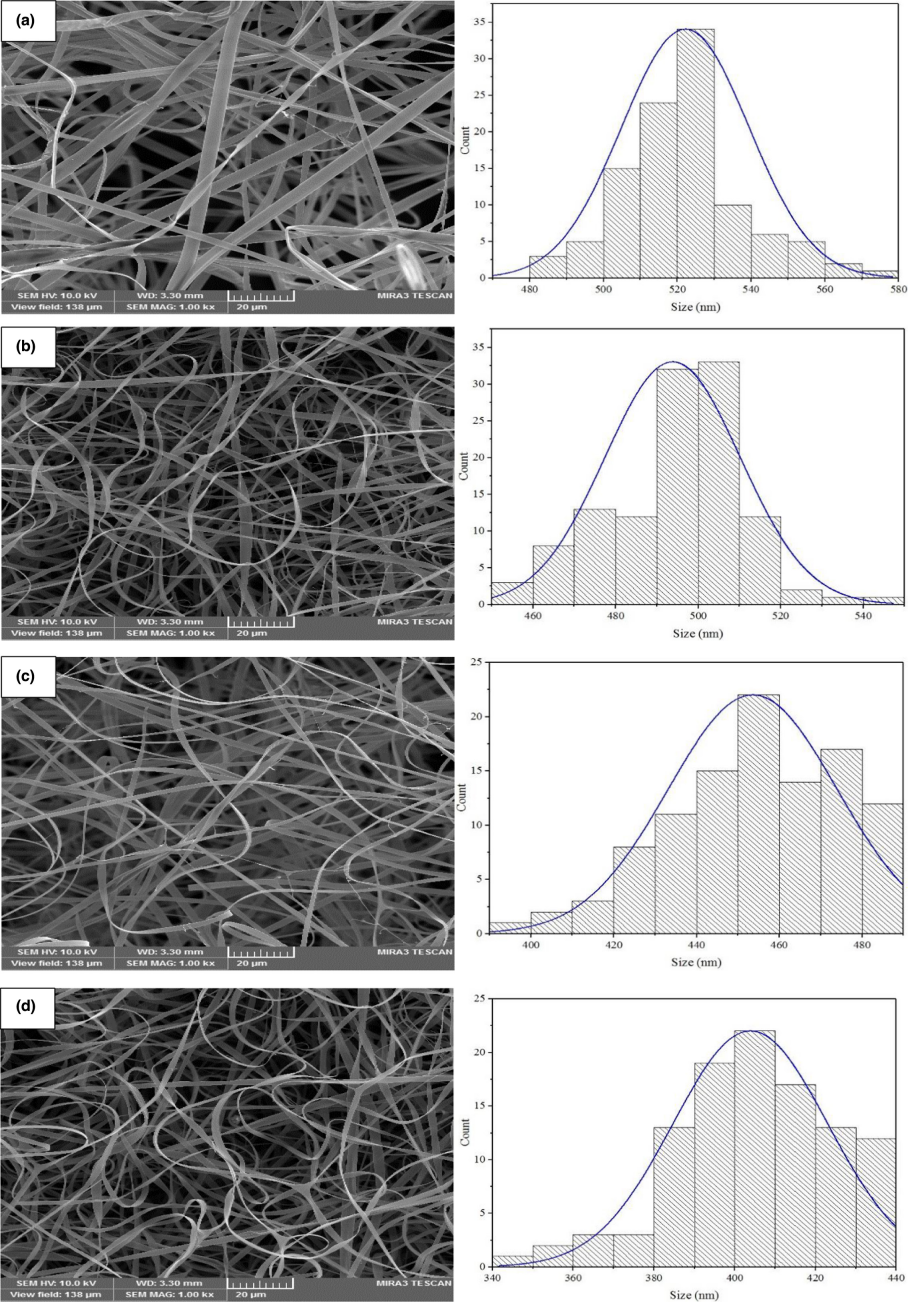
Field emission scanning microscopy (FESEM) of (a) net gliadin mat and gliadin mats containing of (b) 5% *Zataria multiflora Boiss* essential oil (ZMEO), (c) 10% ZMEO, and (d) 15% ZMEO

Figure 2 shows the related results of the morphological properties of gliadin electrospun nanofibers loaded with the ZMEO. Pure gliadin nanofibers showed a uniform and beaded‐free structure with a diameter of 522.19 ± 11.23 nm (Figure 2a). Morphological properties of pure gliadin nanofibers underwent major changes by loading different levels of the ZMEO (5, 10, and 15% W/W). Accordingly, as shown in Figure 2, the samples of electrospun gliadin nanofibers loaded with the ZMEO (Figure 2b–d) had a smaller mean diameter than the control sample of gliadin nanofibers. It was also observed that with increasing the concentration of essential oil from 5 to 15% w/w, the average diameter of nanofibers decreased from 493.81 ± 10.15 nm, 453.56 ± 14.09 nm to 403.87 ± 15.29 nm, respectively. Decreasing the diameter of nanofibers as a result of increasing the concentration of essential oil may be associated with a decrease in the apparent viscosity of the solutions. Due to their oily nature, EOs act as plasticizer between gliadin polymer chains, reducing the viscosity of the solution. Reducing the viscosity in solutions that have the necessary concentration for the electrospinning process causes the solution to have a good opportunity to spin well in the electric field and thus reduce the diameter of the fibers (Mori et al., 2015; Rezaeinia, Emadzadeh, & Ghorani, 2020; Rezaeinia, Ghorani, et al., 2020). Similar results have been reported for reducing the diameter of electrospun zein nanofibers by the EOs of *Laurus nobilis* and *Rosmarinus officinalis* (Göksen et al., 2020) and clove (Tayebi‐Moghaddam et al.,Tayebi‐Moghaddam et al., 2021). The researchers attributed the decrease in the diameter of the electrospun fibers of the zein by increasing the level of application of EOs in connection with the reduction in the viscosity of feed solutions. But, in general, beaded‐free and uniform structures with almost similar pure gliadin mats were successfully produced from gliadin nanofibers containing different levels of ZMEO.

### 3.4 Mechanical properties

The mechanical properties of electrospun mats based on gliadin nanofibers as a result of using different levels of the ZMEO (5, 10, and 15% w/w) and comparing the mean of the data based on Duncan's multirange test are shown in Table 4. For this purpose, the indices of Young's modulus (EM), tensile strength (TS), and elongation at break (%E) were evaluated. Evaluation of mechanical indicators showed that the use of the ZMEO and increasing its level were significantly (*p* < .05) effective on these indicators. Based on this, it was found that the TS and the EM showed similar behavior as a result of increasing the use of the ZMEO. Therefore, using the ZMEO and increasing its level (5 to 15% w/w) significantly (*p* < .05) led to a decrease in the TS and the EM of the mats. The %E also showed inverse behavior with other indicators of the mats. Therefore, as shown in Table 4, by increasing the level of application of the ZMEO from 5 to 15% w/w, the amount of the %E of the mats increases significantly (*p* < .05) from 8.21 ± 1.23 to 20.36 ± 1.21%.

The EM index directly affects properties such as biodegradability and rigidity, and with increasing this index, the degree of rigidity increases. Therefore, due to the plasticizing role of the ZMEO, by increasing the amount of its application, the EM index and the TS of the mats decrease. The plasticizing ability of the ZMEO may increase the mobility of molecular chains in gliadin biopolymers, which will reduce the rigidity and decrease the EM value (Aguirre et al., 2013; Pereira et al., 2019).

In addition, the plasticizing ability of the ZMEO in the structure of electrospun gliadin nanofibers may weaken the intermolecular forces of gliadin–gliadin and increase the %E of the mats. Therefore, increasing the %E of the mats directly affects their flexibility, so increasing the level of the ZMEO application will increase the flexibility of the mats (Aguirre et al., 2013). Changes in the mechanical properties of electrospun mats as a result of the use of the ZMEO were consistent with other findings of researchers in this field. Scaffaro et al. (2020) evaluate the mechanical properties of EM, TS, and %E of poly(lactic acid) (PLA)‐based electrospun mats containing *Thymus capitatus* essential oil. The results obtained by these researchers showed that the use of essential oil and its increase in the structure of electrospun PLA fibers were associated with a decrease in the EM and TS, which was attributed to the plasticizing ability of the essential oil. The %E of the mats was also evident as a result of the increase in the level of essential oil, which the researchers attributed to the increase in the flexibility of the mats.

### 3.5 FTIR analysis

**FIGURE 3:**
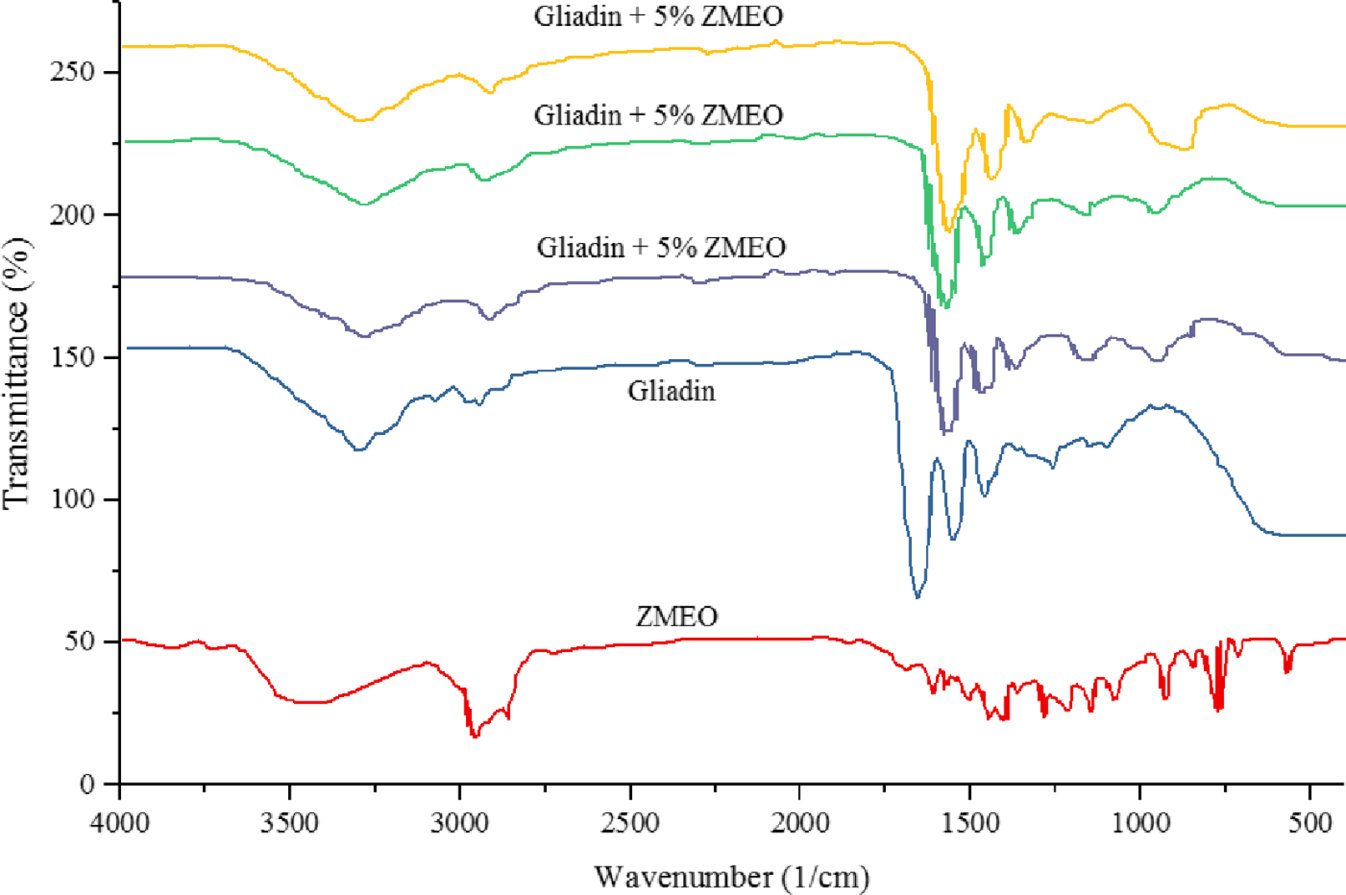
Fourier transform infrared (FTIR) spectrum of net gliadin fibers, pure *Zataria multiflora Boiss* essential oil (ZMEO), and electrospun gliadin fibers loaded with ZMEO

The FTIR analysis of electrospun gliadin mats containing different levels of the ZMEO (5, 10, and 15% w/w) was performed to investigate possible interactions between the components of nanofibers and the essential oil. Therefore, the results of FTIR spectral analysis of net gliadin nanofibers and gliadin nanofibers loaded with different levels of the ZMEO are shown in Figure 3. As the FTIR spectrum of net gliadin nanofibers shows, a broad peak is observed in the region 3296 cm^−1^, which corresponds to the hydroxyl (‐OH) groups of water molecules in the gliadin polymer chain. Also, for net gliadin mats, peaks were observed in the regions of 1652, 1544, 1256, 1098, and 626 cm^−1^, which correspond to amide I, amide II, amide III groups, stretching vibrations of the aromatic ring of the phenylalanine, and vibrations of disulfide bands.

The ZMEO offered different peaks in the range of 4000–400 cm^−1^, the most important peaks of the ZMEO are as follows. Accordingly, the EOs showed peaks in the regions of 3440, 2951, and 2860 cm^−1^ which belong to the hydroxyl groups in the phenolic groups, the stretching vibrations of the asymmetric methyl group, and the bending vibrations of the symmetric methyl group, respectively. Also, in the range of 1600–1400 cm^−1^ vibrating peaks appeared in relation to the C=C‐C rings. In addition, stretching vibrations of aromatic ethers and thymol peaks appeared in 1210 and 843 cm^−1^ areas, respectively (Ardekani et al., 2019).

The effect of loading different levels of the ZMEO on gliadin‐based electrospun nanofibers on changes in position and intensity of net gliadin peaks is shown in Figure 3. Accordingly, as shown in Figure 3, by increasing the loading level, the peaks for the ZMEO became more intense. Hence, the peak was wider in the region 3296 cm^−1^ (hydroxyl group) and lower in frequency (3279 cm^−1^). The frequencies of the amide groups I, II, and III changed to 1617, 1540, and 1179 cm^−1^, respectively. Also, a peak related to thymol (854 cm^−1^) was observed in the structure of all three electrospun gliadin nanofibers loaded with the ZMEO. The presence of peaks related to the ZMEO and gliadin in the structure of the spectrum of gliadin electrospun fibers containing the ZMEO indicates the successful encapsulation of this essential oil in the produced structures. In addition, the change in the position of the gliadin spectrum indicates the interaction of gliadin and the ZMEO. Therefore, due to the hydrophobic nature of gliadin and the ZMEO, hydrophobic interactions probably occur between them (Hosseini et al., 2021; Luo et al., 2011). These findings were consistent with the results of other researchers. Akman et al. (2019) studied the loading of curcumin in the structure of gliadin electrospun nanofibers. Based on the FTIR analysis performed by these researchers, it was found that curcumin was successfully loaded into the structure of electrospun fibers. The study of the FTIR spectrum by these authors showed that with the loading of curcumin, the position of the peaks belonging to the hydroxyl groups, amide groups I, II, and III changes. The researchers attributed the changes in the position and intensity of the peaks as evidence for the successful encapsulation of curcumin in the structure.

### 3.6 DSC analysis

**FIGURE 4:**
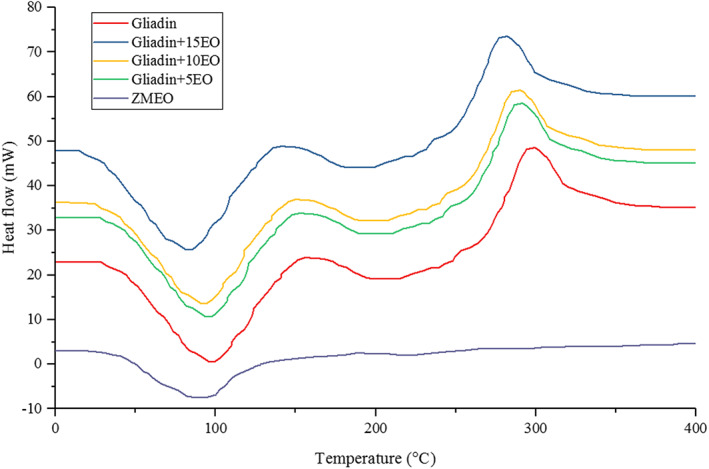
Differential scanning calorimetry (DSC) thermograms for net gliadin fibers, pure *Zataria multiflora Boiss* essential oil (ZMEO), and electrospun gliadin fibers loaded with ZMEO

The DSC analysis was used to evaluate the encapsulation status and thermal stability of the ZMEO in the designed structures. The results of the DSC analysis related to electrospun gliadin nanofibers, pure ZMEO, and electrospun gliadin nanofibers containing different levels of the ZMEO (5, 10, and 15% w/w) are shown in Figure 4. Based on the results of thermal analysis of electrospun gliadin nanofibers, it was determined that its thermogram has two different peaks. In this thermogram, one endothermic peak is observed at 97°C and one exothermic peak at 296°C. Therefore, it can be said that the first endothermic peak is probably related to the evaporation of water molecules, and the exothermic peak may be related to the total degradation temperature of gliadin (Akman et al., 2019). Examination of the thermogram of the ZMEO shows that this essential oil has an endothermic peak at 91°C. This peak is probably related to the evaporation temperature of the essential oil and its volatile parts, which indicates volatility and thermal instability of the ZMEO (Salarbashi et al., 2014).

By loading the ZMEO in the structure of electrospun gliadin nanofibers, changes occurred in the peaks of net gliadin nanofiber and pure ZMEO. Accordingly, as shown in Figure 4, by loading the essential oil (up to 15% w/w) in the structure of electrospun nanofibers, the peak of evaporation of the ZMEO disappeared and the peaks of water evaporation temperature, and their total degradation were transferred to lower temperatures of 83 and 278°C, respectively. These behaviors indicate successful encapsulation and proper loading of the ZMEO in the structure of electrospun gliadin fibers (Rezaeinia, Emadzadeh, & Ghorani, 2020; Rezaeinia, Ghorani, et al., 2020). Therefore, it can be said that the electrospinning process has provided good conditions for encapsulation of the ZMEO in electrospun gliadin nanofibers, which has also increased its thermal stability. Findings of this study are consistent with the results of the DSC analysis of other researchers. Rezaeinia, Emadzadeh, and Ghorani (2020) and Rezaeinia, Ghorani, et al. (2020) loaded menthol crystals into the structure of electrospun gelatin nanofibers and evaluated the thermal properties of the produced nanofibers using DSC analysis. The DSC analysis performed by these researchers showed that menthol is well loaded in the structure of electrospun gelatin nanofibers. They attributed this claim to the disappearance of the peak associated with the dissolution of menthol crystals and the endothermic peak associated with its evaporation in the thermogram of electrospun gelatin nanofibers containing menthol.

### 3.7 Antimicrobial activity

Antimicrobial activity of electrospun gliadin nanofibers with different levels of the ZMEO (5, 10, and 15% w/w) against Gram‐positive (*B. cereus* and *S. aureus*) and Gram‐negative (*E. coli*, and *P. aeruginosa*) bacteria was compared with that of the mats without essential oil (Table 5). Based on the results, it was found that the susceptibility of Gram‐positive bacteria to electrospun mats containing the ZMEO was higher than that of Gram‐negative bacteria. As shown in Table 5, electrospun gliadin mat without essential oil had no antimicrobial effect against any of the studied bacteria, but by increasing the amount of essential oil from 0 to 15% w/w, the diameter of the growth inhibition zone of the studied bacteria increased significantly (*p* < .05). The highest antimicrobial effect (largest diameter of growth inhibition zone) was related to electrospun mats containing 15% w/w of the ZMEO, which was obtained in association with*B. cereus*. Higher sensitivity of Gram‐positive bacteria or in other words more resistance of Gram‐negative bacteria to EOs loaded in the structure of electrospun fibers has been reported by other researchers for electrospinning mats containing clove essential oil (Tayebi‐Moghaddam et al., 2021). They found that *Listeria monocytogenes* and *S. aureus* were more sensitive to the essential oil than *E. coli* and *Salmonella typhimurium*. The researchers attribute this behavior to the multilayer and complex structure of the cell wall of the Gram‐negative bacteria, which contains a peptidoglycan (lipopolysaccharide layer).

**TABLE 5:** Inhibition zone diameter of net electrospun gliadin mat and gliadin mats containing the *Zataria multiflora Boiss* essential oil (ZMEO)

Sample	*B. cereus*	*S. aureus*	*E. coli*	*P. aeruginosa*
Gliadin	0.00 ± 0.00^d^	0.00 ± 0.00^d^	0.00 ± 0.00^d^	0.00 ± 0.00^d^
Gliadin +5% ZMEO	8.23 ± 1.21^c^	9.52 ± 1.09^c^	5.27 ± 1.21^c^	3.42 ± 1.20^c^
Gliadin+10% ZMEO	11.31 ± 1.11^b^	13.41 ± 1.16^b^	8.52 ± 1.22^b^	6.63 ± 1.14^b^
Gliadin+15% ZMEO	15.14 ± 1.13^a^	19.36 ± 1.12^a^	12.32 ± 1.09^a^	10.27 ± 1.21^a^

*Note*: Different letters in the same column indicate significant differences (*p* < .05).

### 3.8 In vitro release modeling of the ZMEO

**FIGURE 5:**
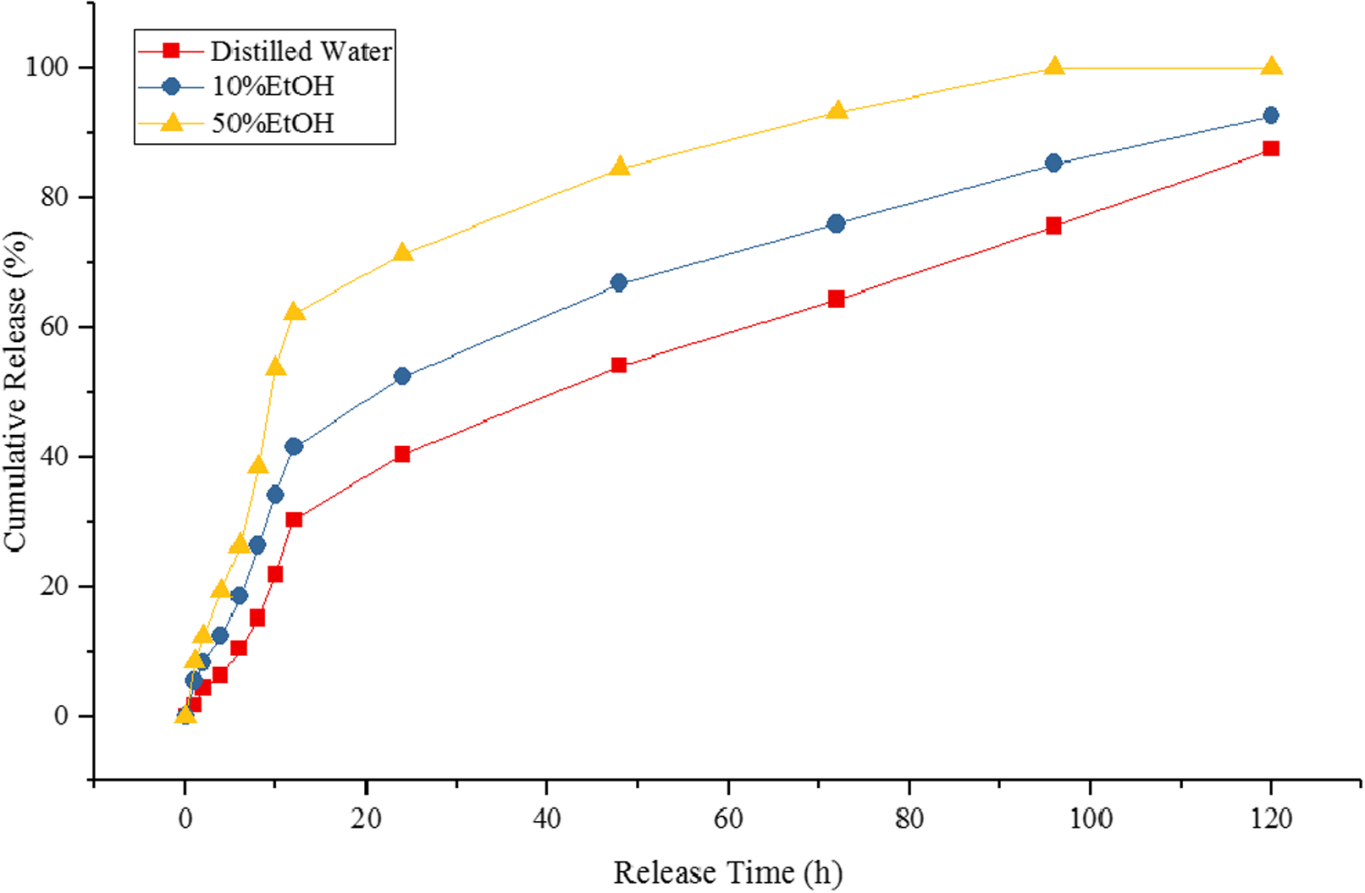
In vitro release profile of the *Zataria multiflora Boiss* essential oil (ZMEO) from electrospun gliadin fiber (containing 15% ZMEO) in various food simulants

Modeling the release of the ZMEO from the structure of electrospun gliadin nanofibers was performed to investigate the release of essential oil in food simulant media and the mechanism governing the release of essential oil from these structures. This is done to be aware of the appropriate media for the controlled release of essential oil and also to identify the target products for the release of essential oil according to the specified purpose. Therefore, different experimental models (Higuchi, Rigter–Peppas, Peppas–Sahlin, Kopcha, and Peleg) were selected to fit the data obtained from the amount of essential oil release in food simulant media (distilled water, 10% ethanol, and 50% ethanol). Based on the results obtained during this study, it was found that in all test media, a rapid and burst release of the ZMEO is observed and then the release is controlled and seen with a gentle slope (Figure 5). It was also observed that the highest release of essential oil in the media consisting of 50% ethanol (simulation of oily food models) and the lowest release of essential oil in the aqueous medium (distilled water as a simulator of water‐based foods) took place. Rapid and immediate release of essential oil in the first short time is probably due to the presence of the ZMEO on the surface of nanofibers and in fact the unencapsulated part, as well as the amount of essential oil that is encapsulated near the surface (Heydari‐Majd, Ghanbarzadeh, Shahidi‐Noghabi, Najafi, Adun, & Ostadrahimi, 2019; Tayebi‐Moghaddam et al., 2021). Also, in the simulated media of fatty foods (50% ethanol), probably due to the greater tendency of gliadin to decompose and dissolve in alcoholic media, it causes gliadin to dissolve in a shorter time in this media, which is accompanied by more release of the ZMEO in this media. However, due to the hydrophobic nature of gliadin, this causes the electrospun nanofibers of gliadin to release their EOs over a longer period of time and show the lowest release rate compared to other media (Xu et al., 2017).

On the other hand, fitting of the ZMEO release data in food simulant media with different experimental models was performed to investigate the dominant mechanism governing the release of the ZMEO in food simulant media from the structure of electrospun gliadin nanofibers. Based on the results, it was found that the best model describing the release behavior of the ZMEO from the structure of electrospun fibers based on gliadin was Peleg model. Accordingly, as shown in Table 6, the Peleg model has the highest *R* ^2^ (0.996) and RMSE = 6.59. According to the studies, if the root mean square error (RMSE) is less than 10, the fit of the data with the desired model will be excellent, but if the RMSE is more than 10, the quality of the fit of the data with the selected model will be reduced (Rezaeinia, Emadzadeh, & Ghorani, 2020; Rezaeinia, Ghorani, et al., 2020).

**TABLE 6:** The cumulative release profile of *Zataria multiflora Boiss* essential oil (ZMEO) from gliadin electrospun nanofibers

Model	Parameters	Distilled water	10% ethanol	50% ethanol
Higuchi	k	7.64	9.00	10.98
	*R* ^2^	0.971	0.971	0.882
	RMSE	5.11	5.39	12.49
Rigter–Peppas	k	5.38	10.67	18.69
	*n*	0.443	0.412	0.375
	*R* ^2^	0.981	0.974	0.932
	RMSE	4.22	5.33	9.89
Peppas–Sahlin	k_1_	4.17	7.77	12.54
	k_2_	0.045	0.165	0.393
	m	0.711	0.635	0.610
	*R* ^2^	0.985	0.986	0.969
	RMSE	3.90	4.14	6.92
Kopcha	A	6.16	10.52	16.70
	B	0.173	0.177	0.670
	*R* ^2^	0.978	0.978	0.958
	RMSE	4.59	4.90	7.73
Peleg	k_1_	0.414	0.224	0.122
	k_2_	0.008	0.009	0.008
	*R* ^2^	0.987	0.990	0.983
	RMSE	3.54	3.17	4.83

Constant parameters of Peppas–Sahlin (k_1_ and k_2_), Kopcha (A and B), and Rigter–Peppas (n) equations were used to describe the dominant mechanism of release of the ZMEO from the electrospun gliadin fiber structure in food simulant media. Based on the results of the analysis of this constant of equations, it was determined that k_1_ > k_2_, A > B, and *n* < 0.45, so it can be concluded that the dominant mechanism on the release of the ZMEO from the structure of electrospun gliadin fibers is the mechanism of the Fickian diffusion or the Case‐I release (Rezaeinia, Emadzadeh, & Ghorani, 2020; Rezaeinia, Ghorani, et al., 2020). The hydrophobic nature of the matrix forming the encapsulant (gliadin) tends to form hydrophobic bonds with 50% ethanol (hydrophobic–hydrophobic) and this causes the electrospun gliadin mats containing the ZMEO to dissolve more quickly in this simulant media and release their loaded essential oil late (Tayebi‐Moghaddam et al., 2021). As the polarity of the media increases, the tendency to dissolve in it decreases and the release time of the essential oil increases. Alinaqi et al. (2021) evaluated the modeling of clove essential oil release from the structure of electrospray nanoparticles in food simulant media. Based on the results obtained by these researchers, it was found that the best model describing the release behavior is the Peleg model and the Fickian diffusion was the dominant mechanism in the release of EOs from these structures.

The results of DSC and FTIR analyses can be related to release. DSC analysis can show both melting temperature and glass transition temperature. Also, the degree of crystallization can be obtained from the enthalpy (area under the curve). As the melting temperature rises, the crystals become more complete and the diffusion decreases. Diffusion from crystalline areas is far less than amorphous areas, and therefore, a higher degree of crystallization means less release. Also, if the glass transition temperature of the nanofiber is lower than room temperature (for example, 10°C), it indicates that the nanofiber is in an amorphous rubbery state at room temperature, and as a result, the release will be higher. Also, if the glass transition temperature is greater than the room temperature, it will indicate that the glass is in an amorphous state and, as a result, the release will be lower. In general, the possibility of forming a crystalline form in electrospun nanofiber is much less than the amorphous state (rubber or glass). In relation to the relationship between the FTIR test and the release, it should be mentioned that FTIR shows whether chemical bonds have formed between the nanofiber and the capsule material or only physical entrapment has occurred. In the first case, the release will be less.

## 4 CONCLUSION

In this study, the ZMEO was loaded in the structure of electrospun gliadin nanofibers and the physicochemical, microbial properties and in vitro release modeling of the essential oil from these structures were investigated. The study of rheological properties showed that the electrospinning feed solutions had a shear‐thinning behavior and the power law model well described their rheological behavior. Morphological characteristics of electrospun nanofibers showed that the diameter of nanofibers decreases by increasing the level of the ZMEO. FTIR and DSC tests proved well that the ZMEO are trapped in the designed structures. Thermal analysis of nanofibers loaded with the ZMEO showed that the use of gliadin as an encapsulant matrix will increase the thermal stability of this essential oil. Release modeling also showed that the best model describing release behavior is the Peleg model. In addition, modeling in food simulant media showed that the dominant mechanism in the release of the ZMEO is the Fickian diffusion mechanism.
